# The adult *HNRNPH1::ERG* positive acute myeloid leukemia with clear lower remission and worse prognosis: A case report and review of the literature

**DOI:** 10.1097/MD.0000000000041809

**Published:** 2025-04-04

**Authors:** Yanyan Lu, Rui Wei, Jianlan Li, Lianrong Xu

**Affiliations:** a Department of Hematology, Second Affiliated Hospital of Shanxi Medical University, Taiyuan, Shanxi, China; b Department of Laboratory of Experimental Diagnostics, Second Affiliated Hospital of Shanxi Medical University, Taiyuan, Shanxi, China.

**Keywords:** AML, *ERG*, *FUS*, *HNRNPH1*

## Abstract

**Rationale:**

Acute myeloid leukemia (AML) derived from *t*(5;21)(q35;q22) translocation, post-transcriptional translation, forming the HNRNPH1::ERG fusion gene is a rare group of recurrent chromosomal abnormality myeloid malignancies. Only 1 adult case of AML has been reported so far. Here we identified a disparate adult case of HNRNPH1::ERG positive AML with clear breakpoint locations by utilizing The RNA sequencing(RNA-seq) and we addressed the clinical, treatment, pathological and molecular mechanism, along with a review of the literature.

**Patients concerns:**

A 54-year-old man visited our department with fever and fatigue for 10 days.

**Diagnoses:**

Diagnosed with acute myeloid leukemia (AML) through morphology, immunology, Cytogenetics, and Molecular biology (MICM) typing, with a confirmed HNRNPH1-ERG fusion gene.

**Interventions:**

Multiple induction chemotherapy combined with targeted therapy was performed.

**Outcomes:**

He died in February 2024.

**Lessons:**

In our review, Only 1 adult case of AML has been reported so far. To summarize the 5 cases in the studies, the HNRNPH1::ERG positive AML cases had a significantly higher blast cell counts and more frequently companied with rare gene mutations, which characterized poorer prognosis and lower remission in adult HNRNPH1::ERG positive AML.

## 1. Introduction

Acute myeloid leukemia (AML) is a hematologic malignancy characterized by abnormal proliferation and impaired differentiation of myeloid hematopoietic stem cells.^[1]^ In recent years, more and more rare fusion genes have been discovered.^[2,3]^ Based on the distribution of cytogenetically and molecularly defined subsets of AML presenting in adults in WHO 5th classification, the rare fusions accounted for 1% of all subtypes, including *t*(16;21)(p11;q22)/*FUS::ERG*.^[4]^

**Figure 1.**
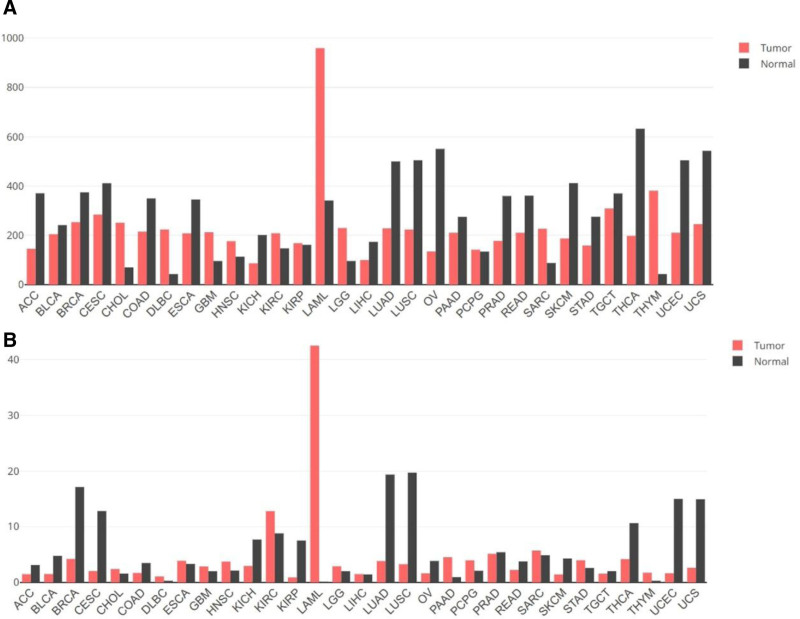
(A, B) The HNRNPH1 gene and ERG gene expression profile across all tumor samples and paired normal tissues. The height of bar represents the median expression value of concerned tumor type or normal tissue. The black bar represents normal tissues, the red bar represents tumor tissues. The median value is 959.67 in tumor samples of the HNRNPH1 gene. (B) The median value is 42.51 in tumor samples of the ERG gene. ACC = adenoid cystic carcinoma, BLCA = bladder urothelial carcinoma, BRCA = breast invasive carcinoma, CESC = cervica squamous cell carcinoma and endocervical adenocarcinoma, CHOL = cholangiocarcinoma, COAD = colon adenocarcinoma, DLBC = lymphoid neoplasm diffuse large B-cell lymphoma, ESCA = esophageal carcinoma, GBM = glioblastoma multiforme, HNSC = head and neck squamous cell carcinoma, KICH = kidney chromophobe, KIRC = kidney renal clear cell carcinoma, KIRP = kidney renal papillary cell carcinoma, LAML = acute myeloid leukemia, LGG = brain lower grade glioma, LIHC = liver hepatocellular carcinoma, LUAD = lung adenocarcinoma, LUSC = lung squamous cell carcinoma, OV = ovarian serous cystadenocarcinoma, PAAD = pancreatic adenocarcinoma, PCPG = pheochromocytoma and paraganglioma, PRAD = prostate adenocarcinoma, READ = rectum adenocarcinoma, SARC = sarcoma; SKCM = skin cutaneous melanoma, STAD = stomach adenocarcinoma, TGCT = testicular germ cell tumors, THCA = thyroid carcinoma, THYM = thymoma, UCEC = uterine corpus endometrial carcinoma, UCS = uterine carcinosarcoma.

**Figure 2.**
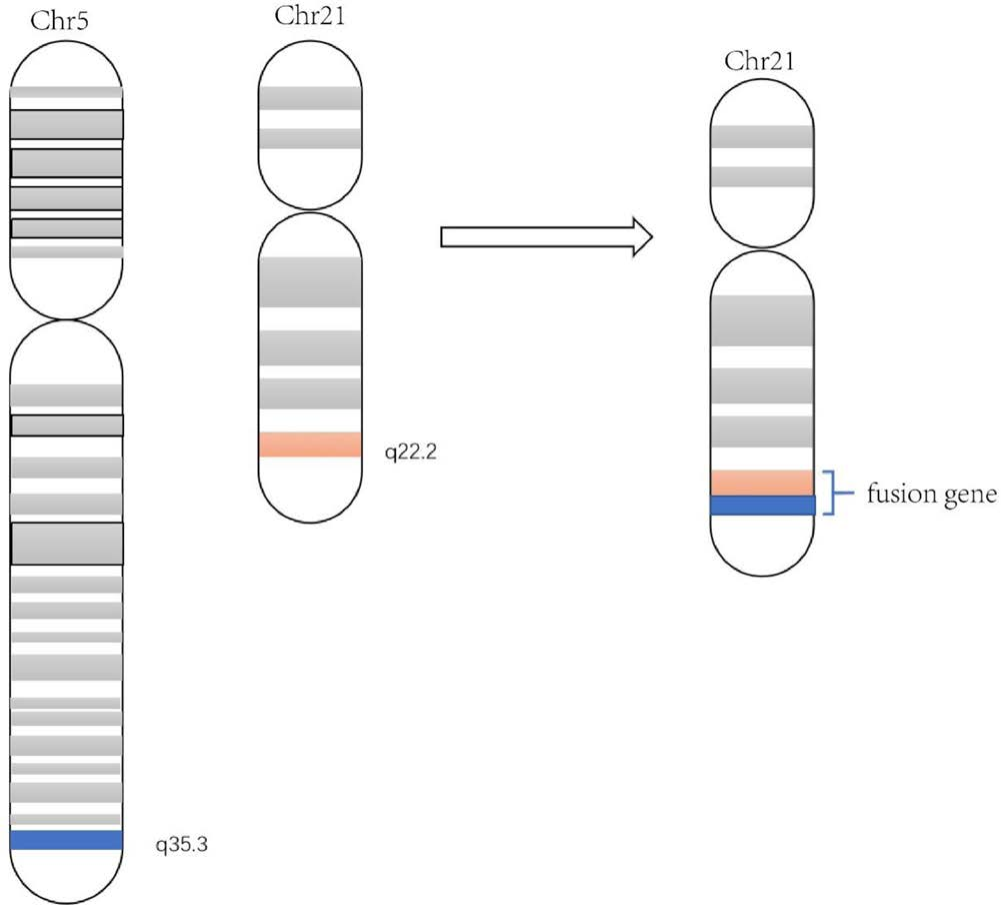
The mutual translocation between 5q35.3 and 21.q22. The blue bar depicts the q35.3 portion on chromosome 5 and the orange bar represents the q22.2 portion on chromosome 21. Both bars contribute to the fusion of *HNRNPH1::ERG* gene partly or wholly.

*HNRNPH1* is located in chromosome band 5q35.3 (*chr5:179,614,178-179,634,784, by genecards*) and contains 13 exons, producing several alternative spliced transcripts.^[5]^ It has been demonstrated that variation 2 is related with poorer tumor differentiation.^[6]^ *ERG* gene is located in chromosome band 21q22.2 (*chr21:38,367,261-38,661,783, by genecards*) and comprises 12 exons encoding 2 isoforms, *ERG*1 and *ERG*2.^[7,8]^ According to GEPIA2 analysis, the *HNRNPH1* and *ERG* gene is most highly expressed in AML^[9]^ (Fig. 1A, B). The *HNRNPH1::ERG* fusion gene deriving from a cytogenetic translocation, leading to the rearrangement of *HNRNPH1* and *ERG* gene, results in AML^[10]^ (Fig. 2). This group’s clinical features are unclear, and the prognosis is equivocal. Reviewing the literature, we found only 1 adult case of *HNRNPH1-ERG* positive AML had previously referred.^[10]^ Herein, we present 1 distinctive case and conducted a literature review summarized in Table 1.^[10–12]^ All participants gave written informed consent before enrollment in the study, which was conducted in accordance with the principles of the Declaration of Helsinki.

**Table 1:** Characteristics of *HNRNPH1-ERG* positive cases referred in the literature.

Number	Sex	Age	WBC ×10^9^/L	Hb ×g/L	PLT ×10^9^/L	BM (%)	Immune phenotype	Karyotype	Gene mutation	Induction chemotherapy	CR	Survival time (mo)	Reference
1	F	42	1.42	81	213	82	80.7% cells with CD117^+^, CD33^+^, CD13dim, HLA-DR^−^, CD64^−^, CD4^−^, CD36^−^, 56, CD19^−^, CD7^−^, CD5^−^, CD61^−^, CD71^−^	No split phase	CSF1R, DDX41	Standard 7 + 3 chemotherapy, with cytarabine 120 mg/m^2^ on days 1–7	No	3	Jiang et al^[10]^
2	M	54	2.66	74	117	55.50	71.6% cells with CD34, CD33, CD123, CD64, CD133, CD7dim, CD38^+^	NR	PHF6, PRPF8	IAC chemotherapy with idarubicin 10 mg/m^2^ on days 1–3, cytarabine 100 mg/m^2^ on days 1–7 and CTX 1000 mg on day 7	No	8	
3	F	7	7.7	NR	NR	96	NR	46, XX[20]	CCND3, ETS2	AAML0531chemotherapy	Yes	18	Bolouri et al^[11]^
4	F	9	16.2	NR	NR	78	NR	46, XX[20]	NR	AAML0531 chemotherapy	Yes	60	Bolouri et al^[11]^
5	M	4	62.7	87	37	90	85% cells with CD34, CD117, CD13 dim, CD33, Cd38 decreased, HLA-DR decreased, CD64 (partial), CD11b (subset), CD15, CD7 (small subset), CD56 (small subset) and MPO (small subset)	NR	NR	AAML1831 chemotherapy	Yes	6	Hainaut et al^[12]^

BM = bone marrow, CR = complete remission, NR = no report, PB = peripheral blood.

## 2. Case report

### 2.1. Patient information

A 54-year-old male was admitted to the hospital on June 2, 2023 with fever and fatigue for 10 days. He had no family history. He was unemployed, with no toxic contact, alcohol, tobacco or other bad habits.

### 2.2. Physical examination and diagnostic assessment

**Figure 3.**
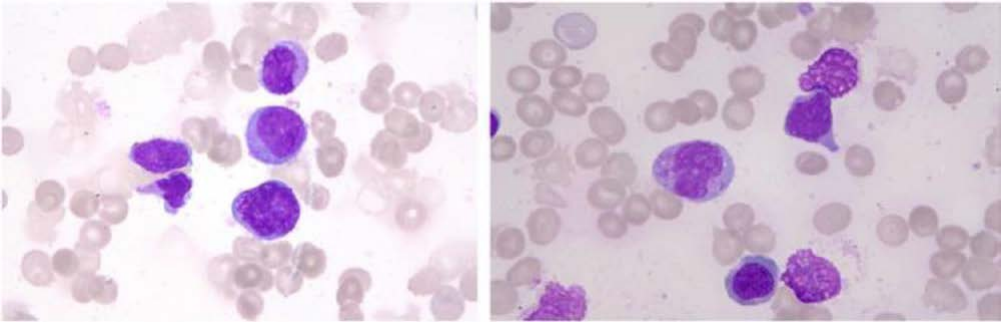
The morphology of bone marrow smear (Giemsa stain, magnification 1000×) shows blast cells ranged from medium to large, replacing normal bone marrow cellularity, which have blue medium or small plasma and some have purple-red granules, and visible pseudopods. The nucleus is polymorphic, such as round, oval, and the nucleolus is large and well-defined.

The peripheral blood (PB) cell count showed the leukocyte (WBC) 2.66 × 10^9^/L, the hemoglobin (HGB) 74 g/L, and the platelet (PLT) 117 × 10^9^/L. Bone marrow (BM) smears showed blast cells accounting for 55.5% of the total (Fig. 3). Flow cytometry showed 71.6% of blasts expressed CD34, CD33, CD123, CD64, CD133, CD7dim, CD38^+^; and did not express CD117, CD19, CD10, CD13, CD56, CD16, CD15, CD14, CD11b, HLA-DR, CD36, cTDT, CD10, CD5, CD2, sCD3, CD4, CD8, CD303, cyCD79a, cyCD3, cyMPO; bone marrow biopsy (BMB) shows heterogeneous proliferation of large, rounded nucleus cells that are less cytoplasmic. The majority of granulocytes were positive for myeloperoxidase (MPO), CD34, CD117 examined by immunohistochemically (IHC). No split phase was detected for the chromosomes. The results of next-generation-sequencing (Supplement file 1, Supplemental Digital Content, http://links.lww.com/MD/O483) showed that the variant allele frequency (VAF) of PHF6 was 3% at class I loci, 3.7% at class II loci, and the VAF of PRPF8 was 5.4% at class II loci. The *HNRNPH1::ERG* fusion gene was detected through RNA-seq.

### 2.3. Therapeutic interventions

This patient was treated with standard IA inductive chemotherapy, with idarubicin 10 mg/m^2^ on days 1 to 3, and cytarabine 100 mg/m^2^ on days 1 to 7.^[13]^ On the seventh day, the BM smear showed that 16.0% of blast cells, and CTX 1000 mg was added for inductive chemotherapy. On the 21st day after chemotherapy, the BM smear revealed 5.0% of blast cells, and the flow cytometry showed 22.79% of blast cells with an immunophenotype of CD34^+^, CD117^−^, CD38^+^, CD7^+^, CD123^−^, CD33^+^, CD13^−^, CD10^−^, HLA-DR^−^, indicating that the patient did not achieve complete remission (CR).^[13]^ Unfortunately, he refused allogeneic hematopoietic stem cell transplantation (allo-HSCT). The patients got the second standard IA plus venetoclax (400 mg on days 1–7) reinductive therapy, but did not receive CR. Then, he got 2 cycle therapy composed of intermediate-dose Cytarabine (2.5 mg/m^2^), and HA (Homoharringtonine 2 mg/m^2^ on days 1–7, and Cytarabine 100 mg/m^2^ on days 1–7) plus Retinoids (40 mg on days 18–30) and Chidamide (30 mg, 2 times/week on days 8–33) respectively.

### 2.4. Follow-up and outcomes

Regrettably, he was still not in CR. He died in February 2024. The overall survival (OS) is 8 months.

## 3. Discussion

**Figure 4.**
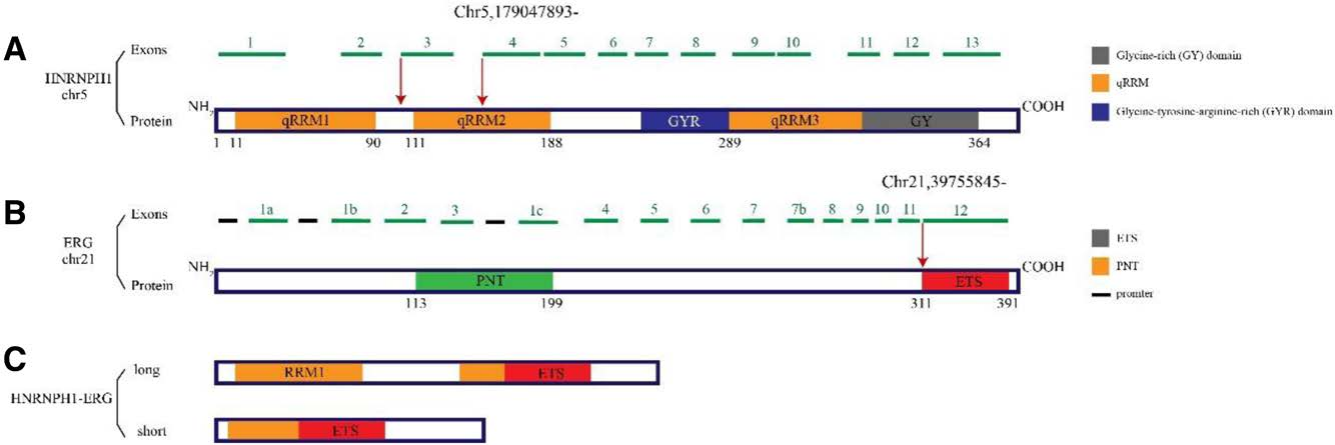
Molecular structure of the *HNRNPH1::ERG*. (A) The molecular structure of heterogeneous nuclear ribonucleoprotein H1 (*HNRNPH1*). The green horizontal line represents the exon of *HNRNPH1* gene. The orange, blue, and gray bars represent different structural domains contained in the protein encoded by the gene, that is, quasi RNA recognition motifs (qRRM1,2,3), glycine-tyrosine-arginine-rich (GYR) domain, and glycine-rich (GY) domain, respectively. (B) The molecular structure of ETS-related gene (*ERG*) protein. The green horizontal line represents the exon of *ERG* gene and the black horizontal line represents the promoter of *ERG* gene. The green and red bars represent the structural domains contained in the protein encoded by the gene, respectively, *ETS* DNA-binding domain (EBD), pointed domain (PNT). (C) The 2 different *HNRNPH1::ERG* fusion transcripts can be generated, including the long and short isoforms. Red arrows represent breakpoint locations. The specific breakpoint locations of our sample are located at the chr5,179047893- of HNRNPH1 and the chr21,39755845- of *ERG* in the figure. EBD = *ETS* DNA-binding domain, GY = glycine-rich, GYR = glycine-tyrosine-arginine-rich, PNT = pointed domain.

Variants of the translocation *t*(5;21)(q35;q22), as well as fusion between *HNRNPH1* and *ERG*, are rarely acquired genetic abnormalities in leukemias.^[10]^ The isoform 2 encoded by the *HNRNPH1* contains 5 RNA-binding structural domains, that is, 2 auxiliary domains (rich in glycine) and 3 quasi RNA recognition motifs (quasi-RRMs).^[14]^ *HNRNPH1* is primarily responsible for causing alternative splicing(AS) in embryonic stem cells (ESCs) to induce differentiation by attaching to the transcription factor TCF3.^[15]^ *HNRNPH1* is a key regulator of RNA maturation, mainly serves in transcription regulation and RNA metabolism.^[5,16]^ *ERG* is a member of the E-26 transformation specific (ETS) family of transcription factors, whose isoform comprises mainly a specific DNA-binding domain called the ETS DNA-binding domain (EBD) and the pointed (PNT) domain.^[17]^ One of the transcription factors in the ETS family, which is strongly linked to hematopoietic stem cells (HSC) self-renewal and the maintenance of differentiation functions, is encoded by the *ERG* gene.^[18]^ It is recognized *ERG* is an oncogene dysregulated overexpression in solid and hematologic malignancies, usually through gene fusions, such as *EWS::ERG* fusion-positive in Ewing’s sarcoma,^[19]^ *TMPRSS2::ERG* fusion-positive in prostate cancer,^[20]^ *FUS::ERG* in AML.^[21]^ In our case, the fusion was formed by recombination of exon 4 (ENST00000442819.2/NM_005520.2) of the HNPNPH1 gene and exon 10 (ENST00000288319.7/NM_182918.3) of the *ERG* gene (Supplement file 2, Supplemental Digital Content, http://links.lww.com/MD/O483). Therefore, we summarize the structure of the *HNRNPH1::ERG* positive AML primary event in Figure 4.

**Figure 5.**
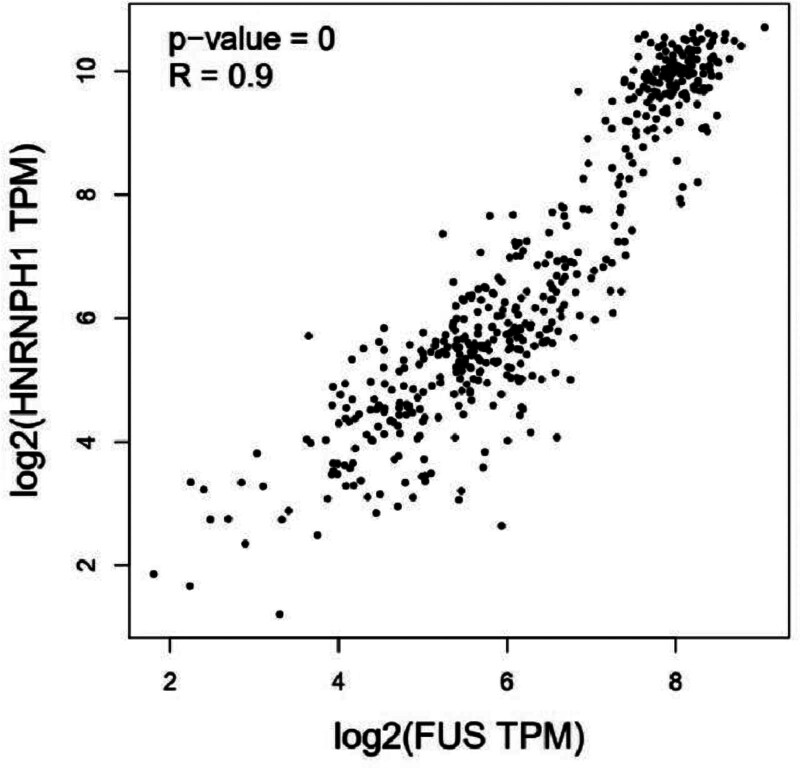
Correlation of *HNRNPH1* gene and *FUS* gene obtained from Gene Expression Profiling Interactive Analysis (GEPIA). *R* represents correlation coefficient. The larger the absolute value of the correlation coefficient, the stronger the correlation. GEPIA = Gene Expression Profiling Interactive Analysis.

**Figure 6.**
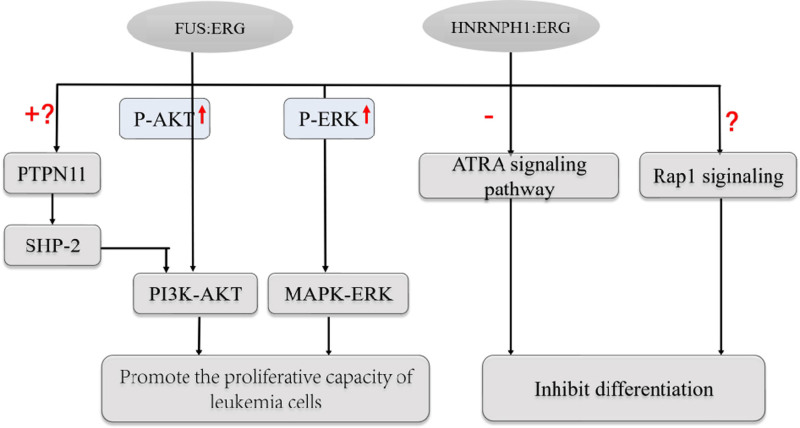
Leukemogenic mechanism of *FUS::ERG* and *HNRNPH1::ERG*. Red “plus” symbol indicates the cooperative relationship; red “minus” symbol represents the inhibitive relationship; red “question” mark represents unclearly detailed regulatory mechanisms. Red “up” arrow represents upregulation. PTPN11 encodes the tyrosine protein kinase SHP-2 which serves as one of the upstream signaling molecules of the PI3K-AKT signaling pathway. Whether PTPN11 mutation frequently co-occur with *FUS::ERG* positivity and the regulatory mechanism is unclear. Nevertheless, it has not yet been discovered that *HNRNPH1::ERG* fusion gene positive and PTPN11 mutation coexist together in AML. The *FUS-ERG* fusion participates in the regulation of the MAPK-ERK and PI3K-AKT signaling pathways by upregulating P-ERK and P-AKT, which promotes the proliferation of leukemia cells. The *FUS-ERG* occupies genomic regions bound by the nuclear receptor heterodimer RXR:RARA, inhibiting the ATRA signaling pathway. The *FUS::ERG* may involve in the Rap1 signaling pathway to inhibit myeloid differentiation, by which mechanism is unclear. The chimeric proteins of *HNRNPH1::ERG* shares the same functional structural domains as *FUS::ERG*, which is considered the same leukemogenic mechanism. AML = acute myeloid leukemia.

The role and possible pathogenic mechanisms of *HNRNPH1::ERG* fusion proteins in AML are currently unknown. Due to the FUS/ETS and *HNRNPH1* commonly belong to the hnRNP family and contain the same RRM functional structural domains,^[14]^ Jiang et al^[10]^ have proposed that AML with *HNRNPH1*:: *ERG* or *FUS::ERG* belong to the same distinct clinicopathologic subtype of AML, that is, AML with *ERG* rearrangement. The high correlation between HNRNPH1 and FUS was verified with an un-*R* value of 0.9 by using GEPIA (Fig. 5).^[9]^ A Chinese study showed that the *FUS::ERG* fusion gene can prevent CD34 cells from myeloid maturation and differentiation, which will cause the cells to stop growing at the stem progenitor and promyelocyte stages.^[22]^ Meanwhile, it has been demonstrated by Xu et al that the *FUS::ERG* fusion gene can participate in the regulation of the MAPK-ERK and PI3K-AKT signaling pathways by upregulating P-ERK and P-AKT, which promotes the proliferative capacity of leukemia cells.^[22–24]^ Furthermore, *FUS-ERG* and the nuclear receptor RARA co-localized in a comparable area, indicating that *FUS-ERG*’s function in leukemogenesis is connected to the ATRA signaling pathway’s suppression.^[25]^ It has been demonstrated that the addition of ATRA reduces *FUS-ERG* binding and promotes AML cell differentiation.^[25]^ It also involves in the Rap1 signaling and PTPN11 pathways.^[22]^ However, the precise regulatory mechanism underlying this phenomenon is still unknown. The *HNRNPH1::ERG* chimeric protein alters the promoter structure of *ERG*^[17]^ and is also highly correlated with *FUS::ERG*. Therefore, we hypothesized that chimeric proteins may have 2 related pathogenic mechanisms in AML: Firstly, the original promoter shape was changed, and normal *ERG* gene expression was not down-regulated, leading to dysregulation of the regulatory network, which regulate 3 major signaling pathways to impact the differentiation and proliferation of myeloid cells (Fig. 6); Secondly, it disrupts the regular metabolism of RNA splicing regulation. A large of fundamental research is required to explore the accurate molecular mechanism.

To summarize the 5 cases, the *HNRNPH1::ERG* positive AML cases had a significantly higher blast cell counts and more frequently companied with rare gene mutations, which characterized poorer prognosis and lower remission in adult *HNRNPH1::ERG* positive AML. The median OS of these 4 cases was 15 months. Age at diagnosis ranged from 4 years old to 54 years old, with a mean age of 9 years old. These features are consistent with the clinical features exhibited by *FUS::ERG* fusion gene-positive AML.^[21]^ Moreover, we attempt to compare the difference between these 2 groups. From a morphological perspective, the typical characteristics of *FUS::ERG* positive AML are eosinophilia, micromegakaryocytes, hemophagocytosis, and vacuolation of leukemic cells.^[21]^ In contrast, this typical feature has not yet been seen in the bone marrow of *HNRNPH1::ERG* positive AML cases, which is certainly related to the small number of cases. From a genetics perspective, Jekarl et al^[26]^ reported a median expression level of CD56 of 45.0 (7.8, 87.0)% in *FUS-ERG*-positive AML patients. CD56 has also been clearly defined as a marker of poor prognosis in AML.^[27]^ In contrast, *HNRNPH1::ERG* positive AML did not show CD56 expression among the 2 patients reported so far. Even though it was discovered that this group may have high levels of CD33 and CD34 expression, this is not specific, and more case numbers need to be summarized in order to identify additional markers. Interestingly, we also discovered that children had longer OS than adults, which may be a random phenomenon.

It has been demonstrated that allo-HSCT improves the prognosis of *FUS-ERG* positive AML patients.^[28]^ In our case, neither the conventional standard chemotherapy regimen nor the combination of chemotherapy and targeted agents failed to achieve CR. Therefore, allo-HSCT should be administered for those suitable candidates once leukemia loads reduce.

## 4. Conclusion

We report an adult case of *HNRNPH1::ERG* of AML with a novel breakpoint and summarize the current breakpoint locations of the *HNRNPH1-ERG* fusion gene, and clinical characteristics. We propose a possible pathogenic mechanism for *HNRNPH1::ERG* sharing the same structural domains as *FUS::ERG*, which is thought to belong to the same subtype of AML by comparing the difference between them. And we found that adult HNRNPH1::ERG positive AML patients have a worse prognosis and lower remission rate. Finally, considering the poor prognosis of *HNRNPH1-ERG* positive patients, allo-HSCT is a unique optimal therapy for those fit candidates.

## Author contributions

**Conceptualization:** Yanyan Lu, Rui Wei, Lianrong Xu.

**Data curation:** Yanyan Lu, Rui Wei.

**Formal analysis:** Yanyan Lu, Rui Wei, Lianrong Xu.

**Funding acquisition:** Lianrong Xu.

**Investigation:** Yanyan Lu, Lianrong Xu.

**Methodology:** Yanyan Lu, Rui Wei, Jianlan Li, Lianrong Xu.

**Project administration:** Yanyan Lu, Rui Wei, Jianlan Li, Lianrong Xu.

**Resources:** Yanyan Lu, Rui Wei, Jianlan Li, Lianrong Xu.

**Software:** Yanyan Lu, Rui Wei, Lianrong Xu.

**Supervision:** Yanyan Lu, Lianrong Xu.

**Validation:** Yanyan Lu, Lianrong Xu.

**Visualization:** Yanyan Lu, Jianlan Li, Lianrong Xu.

**Writing – original draft:** Yanyan Lu.

**Writing – review & editing:** Yanyan Lu, Lianrong Xu.

## Supplementary Material


