# Supplementary material for: The adult HNRNPH1::ERG positive acute myeloid leukemia with clear lower remission and worse prognosis: A case report and review of the literature
Source: Medicine (Baltimore). 2025 Apr 4;104(14):e41809. doi: 10.1097/MD.0000000000041809 (PMC11977748; doi:10.1097/MD.0000000000041809)
Supplement: Supplementary file 1 [file medi-104-e41809-s001.docx]

248-clock gene detection for blood tumors:

ABL1 ACTB ALK ANKRD26 APC ARID1A ARID1B ARID2 ASXL1

ASXL2 ASXL3 ATG2B ATM ATRX BAP1 BCL10 BCL11B BCL2 BCL3 BCL7A BCOR BCORL1 BCR BLM BRAF BRCA1 BRCA2 BRCC3 BRD4 BTG1 CAD CALR CBFB CBL CBLB CCND3 CDKN1B CDKN2A CDKN2B CEBPA CHD2 CHEK2 CIITA CMYA5 COL1A1 CPS1 CREBBP CRLF2 CSF3R CSMD1 CTCF CUX1 CUX2 CXCR4 DDX41 DHX15 DICER1 DNMT3A DOT1L DTX1 EBF1 EED EGR1 EP300 EPCAM EPHA7 EPOR ERBB3 ERG ESCO2 ETNK1 ETS1 ETV6 EWSR1 EZH1 EZH2 FAF1 FANCA FANCB FANCC FANCD2 FANCE FANCF FANCG FANCI FANCL FANCM FAS FAT4 FBN2 FBXO11 FBXW7 FGFR1 FGFR3 FLT3 FOXP1 GATA1 GATA2 GATA3 GFI1 GFI1B

GNAS GNB1 GSKIP HDAC4 HRAS HUWE1 IDH1 IDH2 IKZF1 IKZF2 IKZF3 IL6ST IL7R INPP5D JAK1 JAK2 JAK3 JARID2 KDM5C KDM6A KIT KMT2A KMT2C KMT2D KRAS KSR2 LAMB4 LRP1B LRRK2 LUC7L2 MAF MAFB MAP3K1 MAP3K7 MAPK1 MBD4 MDM2 MDM4 MECOM MED12 MEF2B MEF2C MET MGA MLH1 MLLT1 MLLT10 MPL MSH2 MSH3 MSH6 MTOR MYC MYD88 NCOR1 NCOR2 NCSTN NF1 NFE2 NFKB2 NIPBL NOTCH1 NOTCH2 NPM1 NRAS NSD1 NSD2 NT5C2 NTRK3 NUP214 NUP98 PASK PAX5 PBX1 PC PDGFRA PDGFRB PDS5B PHF6 PIGA PIK3CA PIK3R1 PML PMS2 POT1 PPM1D PRDM1 PRPF40B PRPF8 PRPS1 PTEN PTPN11 PTPN2 PTPRD PTPRT RAD21 RARA RB1 RELN RET ROBO1 ROBO2 ROS1 RUNX1 SAMD9 SAMD9L SETBP1 SETD1B SETD2 SF1 SF3A1 SF3B1 SH2B3 SLX4 SMC1A SMC3 SOCS1 SOS1 SRP72 SRSF2 STAG2 STAT3 STAT5B SUZ12 SYK TBL1XR1 TCF3 TET1 TET2 TET3 TNFAIP3 TOP1 TP53 TP63 TPMT TRIM63 TUSC3 U2AF1 U2AF2 USH2A VHL WAS WT1 ZBTB7A ZMYM3 ZNF687

ZRSR2

Supplemental Digital Content 1

**The sequence of HNRNPH1-ERG:**

ERG_HNRNPH1_chr5_179047892_ENST00000442819_ERG_chr21_39755845_ENST00000398897_length(transcript)=4411nt_BP=514nt
GCAGAACTCGCACAAGGGACCTTATTTAGGTTGCGCAGGCGCCCGCTGGCCATTTCGTCTTAGCCACGCAGAAGTCGCGTGTCTAGTTTGTTTCGACGCCGGACCGCGTAAGAGACGATGATGTTGGGCACGGAAGGTGGAGAGGGATTCGTGGTGAAGGTCCGGGGCTTGCCCTGGTCTTGCTCGGCCGATGAAGTGCAGAGGTTTTTTTCTGACTGCAAAATTCAAAATGGGGCTCAAGGTATTCGTTTCATCTACACCAGAGAAGGCAGACCAAGTGGCGAGGCTTTTGTTGAACTTGAATCAGAAGATGAAGTCAAATTGGCCCTGAAAAAAGACAGAGAAACTATGGGACACAGATATGTTGAAGTATTCAAGTCAAACAACGTTGAAATGGATTGGGTGTTGAAGCATACTGGTCCAAATAGTCCTGACACGGCCAATGATGGCTTTGTACGGCTTAGAGGACTTCCCTTTGGATGTAGCAAGGAAGAAATTGTTCAGTTCTTCTCAGGCAGTGGCCAGATCCAGCTTTGGCAGTTCCTCCTGGAGCTCCTGTCGGACAGCTCCAACTCCAGCTGCATCACCTGGGAAGGCACCAACGGGGAGTTCAAGATGACGGATCCCGACGAGGTGGCCCGGCGCTGGGGAGAGCGGAAGAGCAAACCCAACATGAACTACGATAAGCTCAGCCGCGCCCTCCGTTACTACTATGACAAGAACATCATGACCAAGGTCCATGGGAAGCGCTACGCCTACAAGTTCGACTTCCACGGGATCGCCCAGGCCCTCCAGCCCCACCCCCCGGAGTCATCTCTGTACAAGTACCCCTCAGACCTCCCGTACATGGGCTCCTATCACGCCCACCCACAGAAGATGAACTTTGTGGCGCCCCACCCTCCAGCCCTCCCCGTGACATCTTCCAGTTTTTTTGCTGCCCCAAACCCATACTGGAATTCACCAACTGGGGGTATATACCCCAACACTAGGCTCCCCACCAGCCATATGCCTTCTCATCTGGGCACTTACTACTAAAGACCTGGCGGAGGCTTTTCCCATCAGCGTGCATTCACCAGCCCATCGCCACAAACTCTATCGGAGAACATGAATCAAAAGTGCCTCAAGAGGAATGAAAAAAGCTTTACTGGGGCTGGGGAAGGAAGCCGGGGAAGAGATCCAAAGACTCTTGGGAGGGAGTTACTGAAGTCTTACTACAGAAATGAGGAGGATGCTAAAAATGTCACGAATATGGACATATCATCTGTGGACTGACCTTGTAAAAGACAGTGTATGTAGAAGCATGAAGTCTTAAGGACAAAGTGCCAAAGAAAGTGGTCTTAAGAAATGTATAAACTTTAGAGTAGAGTTTGGAATCCCACTAATGCAAACTGGGATGAAACTAAAGCAATAGAAACAACACAGTTTTGACCTAACATACCGTTTATAATGCCATTTTAAGGAAAACTACCTGTATTTAAAAATAGAAACATATCAAAAACAAGAGAAAAGACACGAGAGAGACTGTGGCCCATCAACAGACGTTGATATGCAACTGCATGGCATGTGCTGTTTTGGTTGAAATCAAATACATTCCGTTTGATGGACAGCTGTCAGCTTTCTCAAACTGTGAAGATGACCCAAAGTTTCCAACTCCTTTACAGTATTACCGGGACTATGAACTAAAAGGTGGGACTGAGGATGTGTATAGAGTGAGCGTGTGATTGTAGACAGAGGGGTGAAGAAGGAGGAGGAAGAGGCAGAGAAGGAGGAGACCAGGGCTGGGAAAGAAACTTCTCAAGCAATGAAGACTGGACTCAGGACATTTGGGGACTGTGTACAATGAGTTATGGAGACTCGAGGGTTCATGCAGTCAGTGTTATACCAAACCCAGTGTTAGGAGAAAGGACACAGCGTAATGGAGAAAGGGGAAGTAGTAGAATTCAGAAACAAAAATGCGCATCTCTTTCTTTGTTTGTCAAATGAAAATTTTAACTGGAATTGTCTGATATTTAAGAGAAACATTCAGGACCTCATCATTATGTGGGGGCTTTGTTCTCCACAGGGTCAGGTAAGAGATGGCCTTCTTGGCTGCCACAATCAGAAATCACGCAGGCATTTTGGGTAGGCGGCCTCCAGTTTTCCTTTGAGTCGCGAACGCTGTGCGTTTGTCAGAATGAAGTATACAAGTCAATGTTTTTCCCCCTTTTTATATAATAATTATATAACTTATGCATTTATACACTACGAGTTGATCTCGGCCAGCCAAAGACACACGACAAAAGAGACAATCGATATAATGTGGCCTTGAATTTTAACTCTGTATGCTTAATGTTTACAATATGAAGTTATTAGTTCTTAGAATGCAGAATGTATGTAATAAAATAAGCTTGGCCTAGCATGGCAAATCAGATTTATACAGGAGTCTGCATTTGCACTTTTTTTAGTGACTAAAGTTGCTTAATGAAAACATGTGCTGAATGTTGTGGATTTTGTGTTATAATTTACTTTGTCCAGGAACTTGTGCAAGGGAGAGCCAAGGAAATAGGATGTTTGGCACCCAAATGGCGTCAGCCTCTCCAGGTCCTTCTTGCCTCCCCTCCTGTCTTTTATTTCTAGCCCCTTTTGGAACAGAAGGACCCCGGGTTTCACATTGGAGCCTCCATATTTATGCCTGGAATGGAAAGAGGCCTATGAAGCTGGGGTTGTCATTGAGAAATTCTAGTTCAGCACCTGGTCACAAATCACCCTTAATTCCTGCTATGATTAAAATACATTTGTTGAACAGTGAAC
AAGCTACCACTCGTAAGGCAAACTGTATTATTACTGGCAAATAAAGCGTCATGGATAGCTGCAATTTCTCACTTTACAGAAACAAGGGATAACGTCTAGATTTGCTGCGGGGTTTCTCTTTCAGGAGCTCTCACTAGGTAGACAGCTTTAGTCCTGCTACATCAGAGTTACCTGGGCACTGTGGCTTGGGATTCACTAGCCCTGAGCCTGATGTTGCTGGCTATCCCTTGAAGACAATGTTTATTTCCATAATCTAGAGTCAGTTTCCCTGGGCATCTTTTCTTTGAATCACAAATGCTGCCAACCTTGGTCCAGGTGAAGGCAACTCAAAAGGTGAAAATACAAGGTGACCGTGCGAAGGCGCTAGCCGAAACATCTTAGCTGAATAGGTTTCTGAACTGGCCCTTTTCATAGCTGTTTCAGGGCCTGTTTTTTTCACGTTGCAGTCCT
TTTGCTATGATTATGTGAAGTTGCCAAACCTCTGTGCTGTGGATGTTTTGGCAGTGGGCTTTGAAGTCGGCAGGACACGATTACCAATGCTCCTGACACCCCGTGTCATTTGGATTAGACGGAGCCCAACCATCCATCATTTTGCAGCAGCCTGGGAAGGCCCACAAAGTGCCCGTATCTCCTTAGGGAAAATAAATAAATACAATCATGAAAGCTGGCAGTTAGGCTGACCCAAACTGTGCTAATGGAAAAGATCAGTCATTTTTATTTTGGAATGCAAAGTCAAGACACACCTACATTCTTCATAGAAATACACATTTACTTGGATAATCACTCAGTTCTCTCTTCAAGACTGTCTCATGAGCAAGATCATAAAAACAAGACATGATTATCATATTCAATTTTAACAGATGTTTTCCATTAGATCCCTCAACCCTCCACCCCCAGTCCAGGTTATTAGCAAGTCTTATGAGCAACTGGGATAATTTTGGATAACATGATAATACTGAGTTCCTTCAAATACATAATTCTTAAATTGTTTCAAAATGGCATTAACTCTCTGTTACTGTTGTAATCTAATTCCAAAGCCCCCTCCAGGTCATATTCATAATTGCATGAACCTTTTCTCTCTGTTTGTCCCTGTCTCTTGGCTTGCCCTGATGTATACTCAGACTCCTGTACAATCTTACTCCTGCTGGCAAGAGATTTGTCTTCTTTTCTTGTCTTCAATTGGCTTTCGGGCCTTGTATGTGGTAAAATCACCAAATCACAGTCAAGACTGTGTTTTTGTTCCTAGTTTGATGCCCTTATGTCCCGGAGGGGTTCACAAAGTGCTTTGTCAGGACTGCTGCAGTTAGAAGGCTCACTGCTTCTCCTAAGCCTTCTGCACAGATGTGGCACCTGCAACCCAGGAGCAGGAGCCGGAGGAGCTGCCCTCTGACAGCAGGTGCAGCAGAGATGGCTACAGCTCAGGAGCTGGGAAGGTGATGG
GGCACAGGGAAAGCACAGATGTTCTGCAGCGCCCCAAAGTGACCCATTGCCTGGAGAAAGAGAAGAAAATATTTTTTAAAAAGCTAGTTTATTTAGCTTCTCATTAATTCATTCAAATAAAGTCGTGAGGTGACTAATTAGAGAATAAAAATTACTTTGGACTACTCAAAAATACACCAA

Supplemental Digital Content 2
